# Surgical Informed Consent in Clinical Practice: Patients' Perspective Undergoing Cesarean Section at Three Teaching Hospitals in Addis Ababa, Ethiopia

**DOI:** 10.4314/ejhs.v33i4.13

**Published:** 2023-07

**Authors:** Eskinder Kebede, Tadios Tasew, Dawit worku

**Affiliations:** 1 Department of Obstetrics and Gynecology, CHS, Addis Ababa University, Addis Ababa, Ethiopia; 2 Department of Obstetrics and Gynecology, University Teaching Hospital of Kigali(CHUK), Kigali Rwanda

**Keywords:** Patients' perspective, surgical informed consent, cesarean section

## Abstract

**Background:**

Informed consent is a communication process of providing the patient/parents/guardians with relevant information regarding the diagnosis and the treatment so that they can make informed decisions. This study was to assess the practice of surgical informed consent in Addis Ababa.

**Methods:**

An institution-based cross-sectional study was undertaken in Addis Ababa in 2021. A total of 312 women who underwent cesarean section were interviewed immediately after their hospital discharge. Thirteen components of SIC were used based on international recommendations, including the Royal College of Surgeons' standards of informed consent practices for surgical procedures.

**Results:**

Almost all (100 %) of the respondents were asked to provide written consent, and 96.2 % of them signed the consent form. Most women (89.4%) received information about the indication(s). Few (18.6%) respondents were informed about the type of anesthesia to be administered while only 9 %( n= 28) of them were given an opportunity to choose the option of anesthesia. Only 44.9% of the respondents have received at least six of the 13 components of SIC suggested by the investigators. In this, the most secured data was the signature of the patient which is 96 %. The least documented element of SIC was alternative treatment.

**Conclusion:**

A majority of women who underwent both elective and emergency cesarean section did not receive comprehensive information during the Surgical Informed Consent process in the study hospitals. There is a need that patients need to be counseled during antenatal visits, specifically when patients visit near term for antenatal checkups.

## Introduction

Informed consent is a communication process of providing the patient/parents/guardians with relevant information regarding the diagnosis and the treatment so that they can make informed decisions (1). Surgical Informed Consent (SIC) is permission for a surgical procedure given by an individual who: Is competent, has received all the necessary information, has understood the information, and has given the permission voluntarily (2). Informed consent during surgical practice is an essential component of comprehensive surgical care and is a requirement that should be sought all the time doctor interacts with the patients, although it is very challenging when it comes to implementation (3). Informed consent for medical procedures is a legal requirement in Ethiopia. It was stated that medical service may not be provided without obtaining the patient's informed consent under the Ethiopian Council of Minister's Regulation 299/2013, article 52.

The ultimate goals of SIC are to improve clients' understanding of the intended procedure, and increases client satisfaction. It maintain trust between clients and health providers, and ultimately minimize litigation issues related to surgical interventions.

In obstetrics, explanation of procedures and seeking consent are associated with an improved rating of the birth experience, while non-consented care is seen as an obstacle to skilled birth care utilization(4). Consenting to obstetric interventions including C/S is one of the important elements in the broader concept of respectful maternity care. Several reports have recognized weaknesses in the process of acquiring surgical informed consent for obstetric procedures, such as providing no explanation of the indication for surgery, procedure-related risks or the postoperative trajectory (5, 6).

In obstetric practice, the concept of patient-centered care cannot be fully implemented without institutionalizing a high-quality SIC. Obstetrics is a specialty that is widely perceived to be associated with a high risk of litigation. In the UK, it accounts for about 60–70% of the total (malpractice) sum paid by the NHS Litigation Authority (NHSLA) each year (7). In Ethiopia in seven years (2011–2017), 125 Surgical and medical error claims were reviewed by The Federal Ethics Committee for Health Professionals found that 41/146 (28.1%) of complaints were against Obstetricians and Gynecologists (8).

SIC is the right of a client and the obligation of the health system; however, it is improperly performed and violated in various circumstances (9, 10). Proper SIC can suffer due to health facility factors like lack of a standard consent form, lack of health care provider awareness and experience with SIC, and heavy workload for health care providers (9).

The current status of implementation of informed consent for surgical procedures at the three teaching hospitals in Addis Ababa hospitals are being carried out by the provision of an information sheet, that contains all information that more or less fulfills all criteria for all standard surgical informed consent and the administration of anesthesia to be signed between the patient and the health care provider after the physician informed the procedure to the client. Informed consent adequately satisfies both ethical as well as legal aspects of the standard medical practice (11). It ensures the essential components of consent which include, the patient's autonomy (i.e. freedom of the patient to agree to or to withdraw his consent); patient's competence (i.e. intact cognition to make a deliberate choice); adequate disclosure of all relevant information (including benefits and risks of the planned procedure and of anesthesia); and discussion of the alternative treatments (12).

Globally, there has been growing awareness regarding the profound impact of doctor-patient communication in areas such as consent, and bad news delivery, there is a scarcity of published research on these crucial issues in our country. There is also an apparent lack of realization of their importance among our doctors (13, 14).

The purpose of this study is to assess the current practice of the recommended components of the standard SIC process from patients' perspective, among women undergoing cesarean section at three teaching hospitals in Addis Ababa

## Methods

This is an Institution based cross-sectional study that was conducted from April 1- May 30, 2021, in three teaching hospitals in Addis Ababa, Tikur Anbessa Specialized Hospital, Zewditu Memorial Hospital, and Gandhi Memorial Hospital.

The sample size was determined using a single proportion formula with the level of significance being 5%, Z =confidence level at 95% 1.96, and the absolute precision or margin of error d =0.05. The 26.5% proportion was taken from a study on surgical informed consent done at Hawassa University referral Hospital, OBGYN department. A total of 312 sample size was used. An equal proportion of cases that is 104 cases were used from the three hospitals.

Data collection tools were prepared both in English and in Amharic. The primary versions of the questionnaires were developed through an extensive literature review in English. The questionnaire was translated into Amharic by using standard translating procedures. Data were collected by trained midwives and distributed at the three hospitals based on the sampling proportion. Interviews were conducted after the discharge process was completed. The mean post-op day of data collection was 2.85. Data was collected on discharge from post-cesarean section women who fulfilled the inclusion criteria. Data quality was checked through the careful design of a structured questionnaire and data collection procedure. Pre-test was undertaken on 5 % of the study participants in the post-cesarean section period, at other non-study hospitals. Before the actual data collection started, an amendment was made to the questionnaire.

The inclusion criteria to participate in the research study is “women who had undergone cesarean section and those who gave informed consent”, these women who just had cesarean section and gave their written consent answered the interview questionnaire. The exclusion criteria were women whose cesarean section consent was obtained from a guardian or legal surrogate and Women for whom CS was done at another facility.

The collected data were coded, cleaned, and analyzed using SPSS version 25 statistical software. Descriptive statistics were used to present the result. Tables and different graphs were used to assist in data presentation. Descriptive statistics like frequency and measures of central tendencies were used to describe study participants and to summarize the results of the study. Association of the independent variables with the practice of informed was done using logistic regression and chi-square test; a significant association of variables with outcome was determined using an adjusted odds ratio (AOR) together with a 95 % confidence interval. A variable with a P-value ≤ 0.05 was declared as statistically significant.

### Operational Definition

**Surgical Informed consent (SIC)**: In this study; informed consent related to the process of communication between a physician and patient during the cesarean section and resulted in the patient's agreement and gave written authorization to undergo a cesarean section.

**Practices of informed consent**: The study considers the practice of informed consent of those who had at least 6 components of SIC out of 13 components as a minimum. Thirteen components of SIC were based on international recommendations, including the Royal College of Surgeons' standards of informed consent practices for surgical procedures. Women's satisfaction with the SIC process will be a secondary outcome of interest.

**Patient satisfaction**: Experience score of respondents who give preoperative consent for cesarean section at the three teaching hospital care during the study period

**Health-care provider**: obstetrics and gynecology resident of AAU working at the three teaching hospitals of A.A.

**Ethical considerations**: Ethical approval was obtained from the DRPC of the obstetrics and gynecology department of the College of Medicine and Health Sciences of Addis Ababa University. An official letter was provided to the respective three hospitals administrators. An informed, voluntary, written, and signed consent was taken from each study participant after explaining the purpose, benefits, duration, and any possible risks of the study. The confidentiality of the study participants' information was ensured. Participants' interest to withdraw from the study at any time was assured. No names were recorded in order to keep the identity of respondents anonymous.

## Results

A total of 312 post-cesarean section meeting inclusion criteria from three teaching hospitals in Addis Ababa, Ethiopia, were included in the study, making a response rate of 100%. 39%, (n=122) of respondents were aged between 25 and 29 years. The majority of the respondents are married 98.4%, (n=307) and most of them 41.3%, (n=129) were housewives. About 37.1 % (n=116) had a secondary school education level while 7.7 % (n=24) didn't attend formal education. Orthodox Christianity is the religion to which most respondents belonged to 52.6.4%, (n=164). A majority of the women 92.6 %, (n=289) involved in this study were residing in Addis Ababa.

Nearly 63.8 % of the respondents were multiparous and 34 % had previous cesarean section and 58% had antenatal care follow-up at the facility while only 40.1 %(n=125) got information on the possibility of cesarean section (C/S) at the antenatal clinic. One hundred and ninety-four (62.2%) of the surgeries were emergency C/S, of which 89.7 % (n=174) were in labor and 113 (37.8%) were elective. More than half of the procedures 67.9% (n=212) were done during day time (8 am-18 pm). A majority (96.2%, n=300) of these procedures were performed under spinal anesthesia, while the remaining surgeries were performed under general anesthesia. The majority of the indications for C/S were fetal distress.

Most of the time, consent was given by the patients (93.6%, n=292) and husbands (6.1%, n=19). Two hundred and twenty-four (71.8%) of the respondents were not sure of the cadre of a health care provider who administered the consent process; the remaining participants reported to have received SIC counseling from junior resident physicians 45 (14.4%), senior residents 33 (10.6 %), nurse-midwives 6(1.9%) and medical intern 4 (1.3%).

The majority of the respondents 145(46.5%) had the consent obtained in the labor ward, while 41.0% (n=128) was done in the maternity ward. More than half (55.4%, n=173) of women reported having received SIC counseling immediately prior to their surgery (before the client was put on the operation table), while 0.1 % (n=1) reported having received counseling on the operation table. Meanwhile, 24.7 % (n=77) of women reported that they had received counseling 1 day prior to their surgery, and 19.6 % (n=61) reported having received counseling on the same day. Most counseling language was done in their mother tongue 197(63.1%) and mostly over a period of greater than < 10 minutes (64.7%).

Almost all (100 %) of the respondents were asked to provide written consent, and 96.2 % of them signed the consent form. Most women (89.4%, n=279) reported that they received information about the indication(s) for cesarean section. Most of the women (67.6%, n=211) were not informed about possible alternatives to surgical intervention; 53.4% among elective and 76.3% among emergency clients (p = 0.04). Few (18.6%, n=58) respondents were informed about the type of anesthesia to be administered while only 9 % (n= 28) of them were given an opportunity to choose the option of anesthesia. The majority (76.6%, n=239) said they were not given adequate time for a decision, as well as more than half (66%, n=206) were not given the opportunity to ask questions.

Among the basic components of informed consent, only one-fourth (25 %, n=78) were aware of the benefits of the surgery, and 30.1% (n=100) of them did not know the consequences of refusing the surgery. Only about one-third (34.9 %, n=109) of the patients got information on the risks of undergoing the surgery.

Upon counting the total number of the components of SIC, respondents received before their surgery, 44.9% of the respondents had received at least six of the 13 components of SIC. The mean number of components of SIC received by respondents was computed to be 5.55 with a standard deviation of 2.25.

The associations of the socio-demographic, obstetric, and service-related characteristics of the participants with the receipt of a minimum number of SIC components were examined using a bivariate logistics regression model; strengths of relationships were quantified using Odds Ratio (OR) and 95% confidence interval. Accordingly, previous c/s scar, the literacy level of the respondent, level of formal education, occupation, place of antenatal follow-up, discussion about the possibility of c/s during ANC follow-up, the timing of c/s, time of counseling, a place where informed consent undertaken, easily understand ability of the language used during surgical informed consent, type of surgery, and duration of the time taken by the counselor had an association with receipt of the minimum (at least six) components of SIC (Table 4).

**Table 4:** Essential components of surgical informed consent completed by respondents at GYN/OB. in the three teaching hospitals, Addis Ababa, Ethiopia, 2021

Variable	YES N(%)	NO N(%)
Respondent was asked for surgical informed consent	312(100%)	
Signed on informed consent form	300(96.2%)	12(3.8%)
Given adequate time to make a decision before signing	73(24.4%)	239(76.6%)
Informed about the nature of the procedure	12(4.8%)	297(95.2%)
Informed about reason for cesarean section	279(89.4%)	33(10.6%)
Informed about benefit of surgery	78(25%)	234(75%)
Informed about risk associated with cesarean section	109(34.9%)	203(65.1%)
Given chance to discuss about intended c/s	106(34%)	206(66%)
Informed about type of anesthesia to be used	58(18.6%)	254(81.4%)
Given opportunity to choose from anesthesia options	28(9%)	284(91%)
Given the right to refuse or defer the cesarean section decision	60(19.2%)	252(80.8%)
Informed about the consequences of not undergoing the surgery	212(67.9%)	100(30.1%)

The report of multivariable logistic regression findings indicates that participants with higher education levels had 2.4 times more received the minimum components of surgical informed consent, than illiterate (AOR=2.4, 95%CI=1.81,11.32)., Participants who easily understood the language used during SIC process were 95% received the minimum components of surgical consent than those used non-mother tongue language 46% (AOR=0.06, 95%CI=0.017, 0.23) and, participants with elective surgery received the minimum (at least six) components of SIC 5.9 folds than those with emergency surgery (AOR=5.9, 95% CI=1.08, 31.83, P=0.04). On the other hand, participants who were counseled over more than 5 min were adequately informed, almost 2 folds than those counseled over less than 5 min (AOR=1.8, 95%CI=1.79, 3.69) (Table 5)

**Table 5:** Factors associated with provision of the minimum components of surgical informed consent in the Three Teaching Hospitals, Addis Ababa, Ethiopia, 2021

	Total (N =312)	SIC (N=140)	Adjusted	
Variables	n (%)	n (%)	OR (95%CI)	P
**Parity**				
Primiparous	113(36.2)	46(48.9)	1	0.054
Multiparous	199(63.8)	94(51.1)	0.43(0.18, 1.01)	
**Previous C/S delivery**				
Yes	106(34)	65(46.4)	2.2(0.91, 5.10)	0.08
No	206(66)	75(53.6)	1	
**Timing of Cesarean section**				
08:00-18:00	307(98.4)	112(80)	1.1(0.46, 2.31)	0.929
18:00-8:00	5(1.6)	28(20)	1	
**Type of Cesarean section**				
Elective	118(37.8)	82(58.5)	5.9(1.08, 31.83)	0.04
Emergency	194(62.2)	58(41.5)	1	
**Level of education**				
Primary school	89(28.5)	44(31.4)	1.1(0.33, 3.35)	0.942
Secondary school	116(37.2)	44(31.4)	0.76(0.30, 1.89)	0.554
Higher education	83(26.6)	46(32.8)	2.4(1.81, 11.32)	0.048
Didn't attend	24(7.7)	6(0.04)	1	
**Timing of counseling for collecting consent**				
The day before date of surgery	77(24.7)	50(35.7)		
On the day of surgery	61(19.6%)	35(25)	0.82(0.28, 2.4)	0.724
Few minutes before surgery	173(55.4)	54(38.5)	0.76(0.15, 3.90)	0.737
On the operation table	1(0.3)	1(0.007)		
**Duration of counseling**				
<10 minutes	202(64.7)	75(53.5)	1	0.047
10-30 minutes	110(35.3)	65(46.5)	1.8(1.79, 3.69)	
**Consent collected by**				
Intern	4(1.3%)	2(0.014)		
Junior resident	45(14.4%)	26(18.5)	0.29(0.02, 3.57)	0.338
Senior resident	33(10.6%)	25(17.8)	1.1(1.086, 13.97)	0.945
Nurse-midwife	6(1.9%)	2(0.014)	0.1(0.03,1.36)	0.079
Not known	224(71.8)	85(60.7)	0.30(0.03, 3.17)	0.318
**Understood the language used for counseling**				
Yes	273(87.5)	136(97.1)	1	0
No	39(12.5)	4(2.9)	0.06(0.017, 0.23)	

While the Parity of the respondent, marital status of the respondent, Religion, Grade of the doctor who took the consent, and mother tongue language was not found to be associated with receipt of the minimum (at least six) components of SIC (Table 5).

Patients were asked to assess their level of satisfaction with the informed consent service they received prior to their surgical procedure on a 4-point scale. About one fifth 20.2%, (n=63) of the patients reported that they were very satisfied, (54.2%, n=169 were satisfied), while 17.3% (n=54) were dissatisfied, and 8.3% (n=26) were very dissatisfied with the service.

## Discussion

Obstetric patients present multiple ethical challenges to the healthcare provider at the time of informed consent regarding the disclosure of information. This study reveals that a significant proportion (55.1 %) of women did not receive at least six (the minimum recommendation) of the 13 components of SIC (listed in Table 4) even though almost all (100%) of the respondents were requested for informed consent and signed (96.2%) on the consent form which is nearly similar to the study conducted in Hawassa, Ethiopia (2016) which 99.6 % were asked for informed consent and 99.6 % of them signed on the consent form (15).

This implies surgical informed consent obtained in our setting focused on avoiding legal liability rather than aiding the Patient in decision-making. To say surgical informed consent is valid, the patient needs to accept the surgery willingly after comprehending adequate information about the procedure the nature of the procedure, indication, associated risks and benefits of the procedure, alternative treatment with its risks and benefits, and risk of not having the surgery.

In this study, 89.4% of the patients were told indications for cesarean section. This is comparable with the study in Hawassa, Ethiopia 2016 (15) in which 87% of the patients knew the indication of the procedure.it is also comparable to other different countries' studies (16). In this study even though the majority knew the indication, the aim should be close to 100%. It is unlikely that a patient will know details about the surgery if they do not know the reason for the surgery in the first place.

In this study, participants were not informed of the benefit(s) of their surgeries (75%), often given the opportunity to ask questions (66%), informed about alternative treatment options (76.6%), or counseled concerning the possible complications of the procedure (65.1%). This shows the poor practice of including patients in decision-making. This could be due to physicians' belief that too much information on the details of the procedure might cause excess anxiety in patients, to the extent of leading them to refuse the surgery. Similarly, a study conducted in five hospitals (three public and two private) in Peshawar, Pakistan found that participants didn't receive information on benefits (60%), possible complications on the risk of procedure (53 %), and alternative treatment to surgery (87%) or given a chance to ask questions (68%) (17). A similar study was conducted in Hawassa, Ethiopia, and shows comparable findings (15). On the other hand, a study conducted in the Department of OB/GYN, SRM medical college hospital and research center India found that participants received information on: the benefit of the surgery (95.6%), risk of surgery (94.6%), and alternative treatment (23.7%)(18). Differences among these studies may be due to varying SIC practices, service delivery arrangements, or resource capacities.

Slightly more than half (67.9%) of the patients understood the consequences of refusing the surgery in this study. This number is higher than that was found in a study in Pakistan, where only 9.5 % of patients had this information (19). The higher number here could be due to physicians' attempt to convince the patient to agree to the surgery, by telling them what would happen if they refuse the surgery.

The majority (76.6%) of the patients said they were not given adequate time for a decision in this study. This finding is consistent with the finding of the Hawassa study where 69.1 % of the participants were not given adequate time before signing the consent (15). This higher number could be attributed to the fact that most of the surgeries were emergencies which do not give time to deliberate their decision; other possibilities are the workload at the facility and the knowledge gap on SIC among the residents that need another study. It was found that the majorities of the patients (81.4% & 91% respectively) were not informed about the type of anesthesia and were not given the chance to choose their anesthetic preference. This finding is comparable with other studies (6,18).

Healthcare professionals should introduce themselves (their names and role) before taking the informed consent. In our study, even though 93.6% of the participants believed that they had given their consent for surgery, only 28.2% of the participants knew the identity of the person who obtained consent from them. This highlights the fact that patients in our setting are not conversant with what constitutes informed consent and are not aware of their rights as patients when it comes to decision-making during medical practice. The study found comparable findings with the study in Uganda teaching hospitals which found only 23.7 % of the participants knew the identity of the person who obtained consent from them in spite of the high number (82 %) of the participants who had given their consent for surgery (20).

Despite the poor recall of SIC elements (55.1 %), most patients (74.4%) in this study were paradoxically satisfied or very satisfied with their consent process. Other studies have also reported similar findings (15, 21). This may actually reflect patients' feelings towards their procedure outcome and prognosis, and overall treatment by health care staff rather than just the consent process.

A variety of factors influence the SIC (surgical informed consent) process specifically information transfer and retention, as well as shared decision-making. Low level of education of the patient population, poor communication between doctor and patient, not enough time given for obtaining consent, extensive use of medical terminology, and low level of knowledge of informed consent among doctors. On a structural level, poor working conditions caused by system deficiencies leading to high workloads among practitioners may also add to the problem. In our study, multiple regression analysis shows that the significant factors associated with receipt of the minimum (at least six) components of SIC were the type of surgery, Level of formal education, discussion about the possibility of c/s during ANC follow-ups, and time taken by the counselor during the informed consent process. Other studies have also reported similar findings (15, 21, 22).

Women that had elective surgery received a minimum acceptable number almost two times that of emergency surgery because of the fact that emergency situations in which the informed consent process takes place may not be conducive to information retention due to shortage of time, physical limitations, anxiety, and pain. On the other hand, those counseled over more than five min received minimum components of SIC 1.8 times more than those counseled over less than 5min. the literacy level of the patients is highly associated with comprehension of the provided information. In our study, those who had higher educational levels had received adequate SIC; almost two times that of the uneducated. An antenatal visit is an appropriate time to sensitize a patient about the possibility of a cesarean section. In the study, only 40.1% agreed to the fact that they were told that they might need surgery in the future during their antenatal visit (P=0.001). This finding is comparable with India's study finding that found 37.5 % of participants were told about the possibility the cesarean section during antenatal visits (18).

In conclusion, a majority of women who underwent both elective and emergency cesarean sections did not receive comprehensive information during the SIC process in the study hospitals. This gap in the provision of comprehensive and standard pre-operative counseling for surgical procedures diminishes the overall quality of care clients receive and the ability of the health facility to meet clients' expectations and information needs. Only a small number of the participants were counseled on the possibility of cesarean during antenatal visits.

The problem of proper patient education and information center cannot be tackled only in labor rooms or antenatal wards. Many times, emergent indications warrant immediate action which might not give enough time for proper counseling of patients or their relatives. To bring about awareness about the risks and complications of cesarean section, there is a need that patient to be counseled during the antenatal visits, specifically when patients visit near term for an antenatal checkup. This will help patients actively participate in medical decisions even during emergencies and understand the nature of complications, should they occur.

Moreover, providing educational programs to patients and junior physicians including consent in the curriculum is mandatory to fill the knowledge gaps and thus improve the quality of this process. Adding consent to be checked in the surgical checklist where consent is checked is very important. The consent topic should be considered as one of the CPD refreshers for practitioners.

## Figures and Tables

**Table 1:** Socio-demographic characteristics of respondents at the three teaching hospitals, Addis Ababa, 2021

Variable	N (%)
**Marital status**	
Married	307(98.4)
Others	5(1.6)
**Level of education**	
Primary school	89(28.5)
Secondary school	116(37.2)
Higher education	83(26.6)
Did not attend	24(7.7)
**Occupation**	
House wife	129(41.3)
Government employee	54(17.3)
Private employee	76(24.4)
Private business	44(14.1)
Farmer	2(0.6)
Other	7(2.2)

**Table 2:** Obstetric characteristics of respondents at the three teaching hospitals Addis Ababa, Ethiopia, 2021

Variable	N (%)
**Parity**	
Primiparous	113(36.2)
Multiparous	199(63.8)
**Previous C/S delivery**	
Yes	106(34)
No	206(66)
**Cesarean section decided after the onset of labor**	
Yes	171(54.89
No	23(7.4)
**Type of Cesarean section**	
Elective	118(37.8)
Emergency	194(62.2)

**Table 3:** Circumstance of counseling for collecting informed consent at the three teaching hospitals, Addis Ababa, Ethiopia, 2021

Variable	N (%)
**Timing of Cesarean section**	
08:00-18:00	307(98.4)
18:00- 08:00	5(1.6)
**Consent collected by**	
Intern	4(1.3)
Junior resident	45(14.4)
Senior resident	33(10.6)
Nurse-midwife	6(1.9%)
Specialist	0(0)
Not known	224(71.8)
**Timing of counseling for collecting consent**	
The day before date of surgery	77(24.7)
On the day of surgery	61(19.6)
Few minutes before surgery	173(55.4)
On the operation table	1(0.3)
**Duration of counseling**	
<10 minutes	202(64.7)
10-30 minutes	110(35.3)
**Understood the language used for counseling**	
Yes	273(87.5)
No	39(12.5)
